# Notes and News

**Published:** 1893-03-25

**Authors:** 


### Notes and News.

COLLECTED AND COMMUNICATED.

Upwards of £550 has already been subscribed towards the memorial to Sir Richard Owen, which is to take the form of a marble bust, to be placed in the British Museum. Of this sum H.R.H. the Prince of Wales has contributed £25. More than 300 illustrious names stand on the list of the General Committee. Donations may be sent to the Treasurer, Sir W. H. Flower, K.C.B., Natural History Museum, S.W.

It is stated that the British Association meeting will be held at Oxford in 1894, and that Lord Salisbury, Chancellor of the University, has consented to be president. Lord Salisbury has shown his interest both in University and city by his recent visit in aid of the Radcliffe Infirmary, and it is to be hoped that on his return in 1894 he will be rewarded by reviewing the substantial result of his efforts, and that good progress towards the extension of the institution will have been made.

A meeting of the London and Counties Medical Protection Society was held on the 28th ult., Dr. Heron in the chair. A large number of new members and three new districts added, were reported. Examples of the vigilance of the society were given, and it was resolved "that from the facts which have come to the knowledge of the society, it is the opinion that any recognition by the General Medical Council of the diplomas of Harvard and Michigan, leads to serious injustice, and tends to lower the status of the dental profession in England"

The Prince of Wales presided over the annual meeting of the National Lifeboat Institution last week. It is not the first occasion upon which the Prince has given the institution his powerful advocacy. On this occasion his Royal Highness spoke warmly of both aims and work. At no time has there been greater need for a word in due season, for whilst the stations and appliances are increasing, and the system becoming more effective, the expenses are growing in a like manner. The income last year was deficient to meet expenses by £28,000. The Lifeboat Saturday and Sunday Funds, it is to be hoped, will place the insitution on a better footing, whilst increasing the general interest in it.

The first General Council meeting of the new Association for Women Pioneer Lecturers was held on March 8th at Dr. Williams' Library, Gordon Square. The Rev. H. L. Paget was in the chair. Miss Edith Bradley gave a detailed account of the development and progress of the scheme, and speeches were made by the Chairman and Canon Scott-Holland, who expressed cordial approval of the aims of the Association. The latter provides a fresh field for women lecturers, and proposes to cover the ground between elementary education and University extension lectures. The promoters of the scheme are anxious to work in harmony with all existing educational associations. The Council meeting closed with a discussion on the advisability of including technical subjects in the programme, and Miss Forsyth, in a spirited speech, urged that this should be done, instancing Miss Ethel Lamport's course of sanitation lectures as a step in the right direction.

The seventy-sixth annual meeting of the Royal Hospital for Women and Children was held at the Mansion House on March 10th, the Lord Mayor presiding. An account given of the donations and subscriptions received during the year showed that these included a gift of £500 from Baron Hirsch, £250 from Pinder Simpson, Esq, and from the Executors of Mrs. Ballard £100. The Rose Show held by the Lady Mayoress (Lady Evans), in June last, resulted in a net profit of £1,028 9s. 9d. The Lord Mayor, in the course of his speech, said he had many reasons for taking a special interest in this hospital. Not only was it a City charity, first founded in Doctors' Commons in 1816, but from that time up to the present the Lord Mayors of the day have always filled the office of president. The hospital is doing a most useful work, and since its commencement over 750,000 patients have been relieved. An adverse balance shows itself in the accounts, which will, we venture to believe, be speedily removed, seeing that donors will be encouraged by the fact that the testimony of the Sunday Fund was to the effect that it was the most economical children's hospital in London.

Few even of those who are familiar with the intricacies of the City are aware of the existence of Sir John Cass's Foundation School, which has existed since 1710 in a side street at the Eastern extremity of the City. Yet this institution is now a very wealthy educational charity, whose revenues promise to increase to a large extent as time goes on. The school at present educates 220 children, girls and boys, who are provided with uniform and a daily dinner of a substantial description. The Educational Department does not supervise the institution, which is carried on as an ordinary elementary school. A new scheme is now to be developed. The management is henceforth to be vested in the County Council, School Board, and other authorities. The benefits are to be extended to the densely-populated localities outside the City boundaries. Food and clothing are to be distributed in a more restricted manner, but scholars are to be encouraged by the creation of scholarships and exhibitions to places of higher education. The new system embraces a scheme of technical education on the lines of the People's Palace and the Regent Street Polytechnic. So developed, the ancient charity will prove a far greater blessing than can possibly have been foreseen by its worthy founder.

